# Fructo-Oligosaccharides and Pectins Enhance Beneficial Effects of Raspberry Polyphenols in Rats with Nonalcoholic Fatty Liver

**DOI:** 10.3390/nu13030833

**Published:** 2021-03-03

**Authors:** Bartosz Fotschki, Jerzy Juśkiewicz, Adam Jurgoński, Michał Sójka

**Affiliations:** 1 Division of Food Science, Institute of Animal Reproduction and Food Research, Tuwima 10, 10-748 Olsztyn, Poland; j.juskiewicz@pan.olsztyn.pl (J.J.); a.jurgonski@pan.olsztyn.pl (A.J.); 2 Institute of Food Technology and Analysis, Łódź University of Technology, Stefanowskiego 4/10, 90-924 Łódź, Poland; michal.sojka@p.lodz.pl

**Keywords:** ellagic acid, anthocyanin, flavanol, dietary fibre, obesity, liver disorder

## Abstract

In recent years, nonalcoholic fatty liver disorders have become one of the most common liver pathologies; therefore, it is necessary to investigate the dietary compounds that may support the regulation of liver metabolism and related inflammatory processes. The present study examines the effect of raspberry polyphenolic extract (RE) combined with fructo-oligosaccharides (FOSs) or pectins (PECs) on caecal microbial fermentation, liver lipid metabolism and inflammation in rats with fatty liver induced by an obesogenic diet. The combination of RE with FOSs or PECs reduced the production of short-chain fatty acids in the caecum. RE combined with FOSs exerted the most favourable effects on liver lipid metabolism by decreasing liver fat, cholesterol, triglyceride content and hepatic steatosis. RE and FOSs reduced lobular and portal inflammatory cell infiltration and IL-6 plasma levels. These effects might be related to a decrease in the hepatic expressions of PPARγ and ANGPTL4. In conclusion, PECs and FOSs enhanced the effects of RE against disorders related to nonalcoholic fatty liver; however, the most effective dietary treatment in the regulation of liver lipid metabolism and inflammation caused by an obesogenic diet was the combination of RE with FOSs.

## 1. Introduction

It is increasingly accepted that a diet rich in fats, mostly saturated fatty acids, and low in dietary fibre, increases the risk of obesity and thus development of nonalcoholic fatty liver disease (NAFLD) [1,2]. NAFLD has become the most common liver pathology worldwide, affecting an estimated 25%–30% of most populations. Several pharmacological treatments have been proposed for the treatment of NAFLD, but the reported results are inconclusive [3]. A potentially new approach in the treatment of liver metabolic disorders is the consumption of antioxidant-rich foods in general and of polyphenols or fibre–polyphenol complexes in particular. Diets enriched with polyphenols and dietary fibres may present hepatoprotective effects by increasing fatty acid oxidation and modulating oxidative stress, lipid metabolism and inflammation, which are the main pathogenetic factors linked to the progression from simple fat accumulation to NAFLD [4,5]. Most of the studies focus on the health-promoting effects of dietary fibre or polyphenols while there is a lack of information on how supplementation with dietary fibre may affect the hepatoprotective effects of polyphenols, especially against disorders related to the development of nonalcoholic fatty liver (NAFL).

A valuable source of polyphenols is raspberries. These fruits are known as a rich source of dietary antioxidants largely due to their high levels of phenolic compounds, which are primarily comprised of anthocyanins and ellagitannins [6]. In addition to strong antioxidant properties, raspberry polyphenols have also shown other beneficial bioactivities, including anti-inflammation, antimicrobial activity against pathogenic intestinal bacteria, antiproliferation of human cancer cells and improvement of the blood lipid profile [7,8,9]. A study on hepatocytes showed that polyphenols from raspberries may also regulate immunometabolic signals associated with the development of obesity [10].

An important agent that may modulate the health-promoting effects of polyphenols is the activity of the intestinal microbiota [11]. Well-known dietary compounds that considerably affect the microbiota are fructo-oligosaccharides (FOSs) and pectins (PECs). The chemical structure of these nondigestible saccharides is different, although both of them promote increased bacterial production of short-chain fatty acids (SCFAs) in the hindgut and favourably regulate the blood lipid profile [12,13]. Our previous nutritional study on healthy Wistar rats showed that the addition of polyphenolic extract containing mainly ellagitannins to the diet reduced the positive effects of FOSs in the gastrointestinal tract, while parameters of plasma lipid profile were improved [14]. In the development of NAFL, changes in the blood lipid profile play a significant role [15]; therefore, the regulation of this parameter may support preventive treatment against the development of fatty liver.

Due to previous experiments describing the beneficial action of dietary FOSs and PECs [12,16] in the rat caecum and blood lipid profile as well as the regulatory effects of raspberry polyphenols on obesogenic signals in hepatocytes [10], we hypothesised that a dietary combination of FOSs, PECs and raspberry polyphenols would increase the desired beneficial effect against NAFL-related disorders induced by an obesogenic diet in rats. Moreover, PECs and FOSs exert different effects on hindgut microbiota activity [13]; therefore, combination with raspberry polyphenols could modulate hepatic metabolic mechanisms to different extents.

## 2. Materials and Methods

### 2.1. Raspberry Polyphenolic Extract (RE) Production

The polyphenol extract was obtained from frozen raspberry pomace, which was a waste product from the production of raspberry fruit juice of the “Polana” variety. The exact method of juice production is described in Supplementary Material 1 in our earlier publication [10]. After production, the pomace was immediately frozen and stored at −18 °C for 1 month in sealed polyethylene bags.

After thawing, 20 kg of pomace was subjected to a three-stage extraction with a 30/70 (*v*/*v*) acetone–water mixture. The pomace was placed in a polypropylene tank, and 20 L of extractant (solvent/solid ratio of 1:1) was added. After thoroughly mixing the pomace with the extractant, the tank was closed, and static extraction was carried out for 24 h at 20 °C. The extraction mixture had the character of a dense fruit pulp, which after a specified time was pressed in a laboratory screw-press (capacity of 5 L). After pressing in an amount of approximately 18 L, the raw extract was cooled to 4 °C. The extraction residue was subjected to two additional extractions in the same manner as described above. The extracts obtained from the three stages were combined to obtain a total of 55 L of extract and then filtered using the Hobrafit S40N cellulose filter with 5 μm nominal retention (Hobra-Školnik S.R.O., Broumov, Czech Republic). Acetone was removed from the obtained raw extract using a Heidolph Hei-Vap evaporator equipped with an Automatic Hei-Vap Distimatic module (Heidolph, Schwabach, Germany), where a temperature of 60 °C and a pressure of 135 mbar were applied. The acetone-free extract was again filtered using a Hobrafit S40N filter. The filtered extract (approximately 36 L) was then purified on an Amberlite XAD 1600 sorption bed. This process was carried out on a 50 cm long column with an inside diameter of 7.5 cm in eight cycles. One cycle involved applying 4.5 L extract to the column, followed by washing with 10%, 20%, 30%, 40%, 50% and 60% ethanol solutions (2 L each). After the elution process, the column was conditioned with 2 L of 60% ethanol, 2 L of 30% ethanol and 2 L of water before the next cycle. All solutions used for elution were acidified with formic acid to 0.01%. The purification process was carried out by gravity, with the liquid flow in the range of 20–40 mL/min. The richest fractions in polyphenolic compounds (i.e., ellagitannins, proanthocyanidins and anthocyanins) were fractions eluted with 30% and 40% ethanol. These fractions from 8 cycles (32 L) were combined and subjected to an ethanol removal process using a Heidolph Hei-Vap Distimatic evaporator at 60 °C and 125 mbar. After removing ethanol, the solution was further concentrated (60 °C, 72 mbar) to 6 L and freeze-dried (48 h, −36 °C). Finally, 225 g of purified red powder was obtained in this way.

### 2.2. Polyphenolic Analysis

The ellagitannin and anthocyanins in the produced extract were analysed according to the same procedure and using the same apparatus as described in our previous publication [10]. Briefly, the concentrations of ellagitannin and anthocyanins in extracts diluted with methanol (40 mg/10 mL for ellagitannins and 75 mg/10 mL for anthocyanins) were measured using a Knauer Smartline HPLC system with a DAD detector. The ellagitannin separation was performed on a Gemini C18 column (250 × 4.6 mm; 5 μm, Phenomenex, Torrance, CA, USA). Anthocyanins were separated on a Gemini C18 column (150 × 4.6; 5 μm, Phenomenex). Ellagitannin detection was performed at 250 nm, and the content of individual compounds was calculated on the basis of standard curves plotted for ellagic acid (Extrasynthese, Genay, France), lambertianin C and sanguiin H-6, both produced at Lodz University of Technology according to Klewicka et al. [17]. Anthocyanin detection was performed at 520 nm, and the amount of each anthocyanin was calculated from the curve plotted for cyanidin-3-O-glucoside (Extrasynthese, Genay, France). Ellagitannin and anthocyanin identification was performed using an LC–MS apparatus (Q Exactive Orbitrap, Thermo Fisher Scientific, Waltham, MA, USA) under the same conditions as described in a previous publication [10].

The concentration of proanthocyanidins in the extract was analysed by acid-catalysed degradation of polymeric proanthocyanidins in excess of phloroglucinol, as described by Sójka et al. [18]. For phloroglucinolysis, 20 mg of freeze-dried extract was used. The same analysis conditions (column, gradient, phases, and standards) and FD (λex = 278 nm, λem = 360 nm) detector (Shimadzu RF-10Axl) were used to separate the products of these reactions, but a different chromatography system (Shimadzu, Tokyo, Japan), consisting of an LC-20AD pump, a DGU-20ASR degasser, a CTO-10AS thermostat, and an SIL-20AC autosampler, was used. Concentrations were calculated with standard curves of (−)-epicatechin and (+)-catechin for terminal units and (−)-epicatechin-phloroglucinol adduct for extender units. Free (−)-epicatechin and (+)-catechin were measured under the same HPLC conditions as used for the phenolic extracts and were prepared as above (i.e., prepared for determination of ellagitannins, etc.). The average degree of polymerisation was measured by calculating the molar ratio of all flavan-3-ol units (phloroglucinol adducts + terminal units) to (−)-epicatechin and (+)-catechin, which correspond to terminal units. The chemical characterisation of polyphenols extracted from raspberry pomace is presented in Table 1.

### 2.3. Animals and Experimental Design

The feeding experiment was conducted on 32 growing male Wistar rats aged eight weeks. The animals were allocated to four groups of eight rats each. The rats with initial body weights around 165.3 ± 1.43 g were individually housed in metabolic cages (Tecniplast Spa, Buguggiate, Italy) and a controlled environment (a 12-h light-dark cycle, a temperature of 21 ± 1 °C, relative humidity of 50–70% and 20 air changes per hour). For 12 weeks, each group had free access to tap water and was fed a modified version of the semipurified casein diet recommended for laboratory rodents by the American Institute of Nutrition [19]. With respect to the experimental diets, the control high-fat and low-fibre diet contained 23% lard fat and 3% cellulose (H), the experimental diets were a combination of the H diet with 0.64% of RE (HP diet) and the H diet with RE enriched with 3% of fructo-oligosaccharides (HPF diet) or 3% of pectins (HPP diet). The detailed composition of the diets, which were freely available to rats for the entire experimental period, is shown in Supplemental Table S1. The animal protocol was performed in accordance with European Union Directive (2010/63/EU) for animal experiments and the experiment was approved by the local Institutional Animal Care and Use Committee (Permission No. 51/2016; Olsztyn, Poland).

### 2.4. Collection of Biological Material and Analyticla Procedures

During the experiment, rats were monitored for feed intake and body weight (BW). After 12 weeks of experimental feeding, animals were anaesthetised with a mixture of ketamine and xylazine (100 mg and 10 mg/kg BW, respectively) in physiological salt according to the recommendations for anaesthesia of experimental animals. The body fat and lean masses of anaesthetised rats were determined using time-domain NMR Minispec LF90II analyzer (Bruker). After a laparotomy, blood samples were subsequently collected from the vena cava into heparinised tubes and low-speed centrifuged for 10 min at 350× *g* and 4 °C. Plasma samples were stored at −80 °C until assayed. Next, the small intestine, caecum, epididymal fat and liver were removed, weighed and frozen using liquid nitrogen or were used for further treatment. Samples of fresh small intestine and caecal digesta were collected, and the pH values were measured using a microelectrode and a pH/ION metre (model 301; Hanna Instruments, Vila do Conde, Portugal). Malondialdehyde (MDA) was analysed in the liver tissue according to the procedure developed by Botsoglou et al. [20]. The MDA content was determined spectrophotometrically at 532 nm and expressed in µg of MDA per g of liver tissue. Liver lipids were extracted according to the method of Folch et al. [21]. The liver cholesterol and triglyceride concentrations were determined spectrophotometrically in the extracted lipid phase using commercial kits (Cholesterol DST, Triglycerides DST, Alpha Diagnostics, Ltd., San Antonio, TX, USA).

### 2.5. Caecal Microbial SCFAs

Caecal digesta samples after storage at −80 °C were subjected to SCFA analysis using a gas chromatograph (GC) (Shimadzu GC-2010, Kyoto, Japan) according to previously described method [22]. Briefly, caecal samples (0.2 g) were mixed with 0.2 mL formic acid, diluted with deionised water and centrifuged at 7211× *g* for 10 min. The supernatant was loaded onto a capillary column (SGE BP21, 30 m × 0.53 mm; SGE Europe Ltd., Milton Keynes, UK) using an on-column injector. The initial oven temperature was 85 °C and was raised to 180 °C by 8 °C/min and held there for 3 min. The temperatures of the flame ionisation detector was adjusted to 180 °C and the injection port 85 °C. The sample volume for GC analysis was 1 μL. Pure acetic, propionic, butyric, isobutyric, isovaleric and valeric acids were obtained from Sigma Co. (Poznan, Poland); a mixture of these acids was used to create a standard plot and then to calculate the amount of individual acids. To maintain the calibration every fifth GC run of samples contained pure acids.

### 2.6. Plasma Lipid Profile and Inflammatory Markers

The plasma concentration of cholesterol (total and its HDL), triglycerides and the plasma activities of aspartate transaminase (AST), alanine transaminase (ALT), and alkaline phosphatase (ALP) were determined using an automatic biochemical analyzer (Pentra C200, Horiba, Tokyo, Japan). The non-HDL fraction was calculated according to the following formula: TC-HDL-cholesterol. To measure the concentration of interleukin 6 (IL-6), interleukin 10 (IL-10) and tumour necrosis factor α (TNFα) in the plasma, validated rat ELISA kits were used (Single Analyte ELISArray Kit, Qiagen, Frederick, MD, USA).

### 2.7. Liver Histopathology

The liver was flushed with PBS and dried, and the tissue was immersed for 7 days in a 10% solution of buffered formalin. Liver tissues were embedded in paraffin blocks. Paraffin sections (1–2 μm) were cut with a Reichert’s microtome, and tissue fragments were passed through increasing concentrations of alcohol solutions, acetone and xylene (dewaxed). The preparations were stained with haematoxylin and eosin (H&E; Merck, Darmstadt, Germany) according to the method described by Fischer et al. [23]. The tissue sections were evaluated, and images were taken by standard light microscopy using a computer program for image analysis, B-cell and an Olympus BX50 microscope with a digital camera (Olympus Co., Tokyo, Japan). Hepatic steatosis was evaluated as follows: grade 0, no fat; grade 1, steatosis occupying less than 33% of the hepatic parenchyma; grade 2, 34%–66% of the hepatic parenchyma; grade 3, more than 66% of the hepatic parenchyma. For inflammatory cell infiltration: grade 0: none; grade 1, 1–2 foci/field; grade 2, 3–4 foci/field; grade 3, more than 4 foci/field [24]. Ballooning was graded as minimal, mild or marked [25].

### 2.8. Hepatic Gene Expression

qRT-PCR was performed according to the previously described method [26]. Briefly, RNA was extracted from the liver using TRI Reagent solution (Thermo Fisher Scientific) according to the manufacturer’s instructions. β-actin was selected as a reference gene. The levels of peroxisome proliferator-activated receptor gamma (PPARγ), peroxisome proliferator-activated receptor alpha (PPARα), sterol regulatory element-binding protein 1 (SREBP-1c) and angiopoietin-like 4 (ANGPTL4) mRNA expression single tube TaqMan^®^ Gene Expression Assays (Life Technologies, Carlsbad, CA, USA) were used. Amplification was performed using a 7900HT Fast Real-Time PCR System. The mRNA expression levels of PPARγ, PPARα, SREBP-1c and ANGPTL4 were normalised to β-actin. 

### 2.9. Statistical Analysis

The results are presented as the means and the standard errors of the means (SEMs). Statistica software (StatSoft Corp., Krakow, Poland) was used to determine whether the variables differed among the treatment groups. The statistical analysis was conducted using one-way analysis of variance (ANOVA) and Duncan’s multiple range post hoc test. If the variance was unequal, a Kruskal–Wallis ANOVA by ranks was used followed by Dunn’s post hoc test with Bonferroni correction. Differences were considered significant at *p* < 0.05.

## 3. Results

The growth parameters and basic indicators of the gastrointestinal function of rats fed the experimental diets are shown in Table 2. After 12 weeks of experimental feeding, the diet intake, body weight, and fat, lean and epididymal fat contents of rats were comparable among all groups. In the experimental groups, the tissue mass of the small intestine and the pH value of its digesta were at the same level as those of the control H diet group. However, the tissue mass of the caecum considerably increased in the HPF and HPP groups compared to the control H group. Among all experimental groups, only rats from the HP and HPP groups had reduced pH values in the caecum, but the highest acidification was in the HPP group. The combination of RE with different sources of nondigestible saccharides attenuated microbial SCFA production in the caecum. The lowest production of SCFAs in the caecum was reported in the HPF group. This group also had the lowest concentrations of acetic and butyric acid in the caecum. The concentration of caecal PSCFA was reduced in both groups, but the decrease was more pronounced in rats from the HPP group. Nevertheless, in all groups, the experimental diets did not affect the SCFA profile.

**Figure 1:**
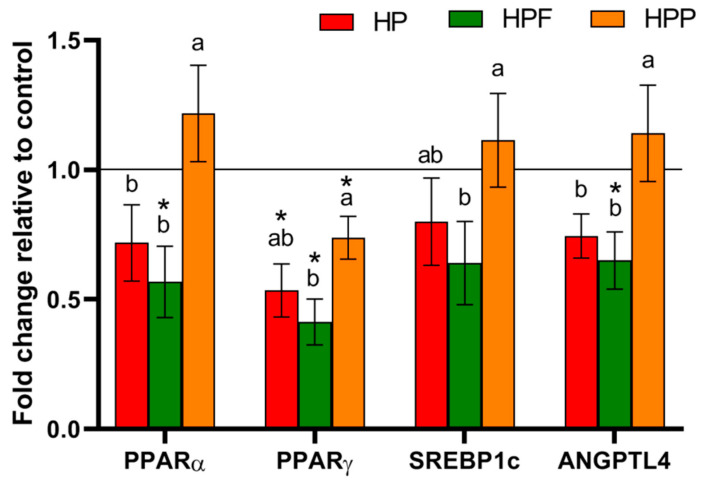
mRNA expression of selected factors associated with lipid metabolism in the livers of rats fed experimental diets. The values are the means ± SEMs. ^a,b^ Mean values not sharing the same superscript letter (a or b) are different at *p* < 0.05 in a post hoc test (Dunn’s test). * Mean values are significantly different in comparison to the control H group, fed a high-fat diet. HP, control high-fat diet enriched with raspberry polyphenol extract; HPF, control high-fat diet enriched with raspberry polyphenol extract and fructo-oligosaccharides; HPP, control high-fat diet enriched with raspberry polyphenol extract and pectin. PPARα, peroxisome proliferator-activated receptor alpha; PPARγ, peroxisome proliferator-activated receptor gamma; SREBP-1c, sterol regulatory element-binding protein 1; ANGPTL4, angiopoietin-like 4.

**Figure 2:**
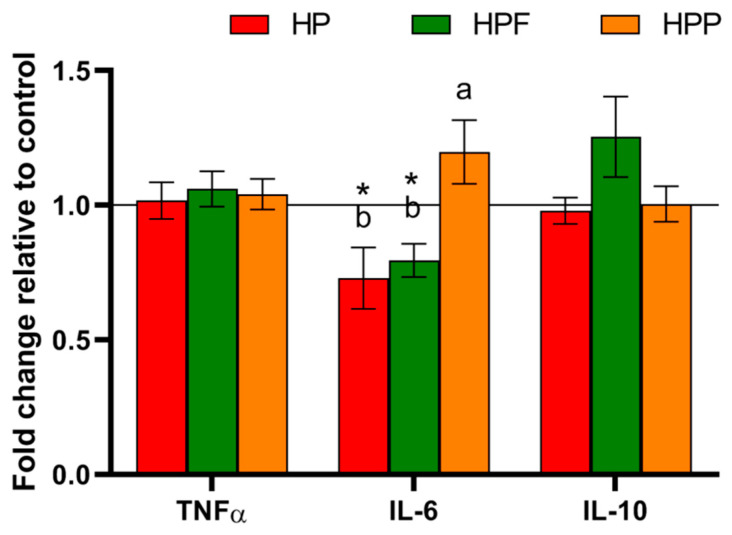
Inflammatory cytokines in the plasma of rats fed the experimental diets. The values are the means ± SEMs. ^a,b^ Mean values not sharing the same superscript letter (a or b) are different at *p* < 0.05 in a post hoc test (Dunn’s test). * Mean values are significantly different in comparison to the control H group, fed a high-fat diet. HP, control high-fat diet enriched with raspberry polyphenol extract; HPF, control high-fat diet enriched with raspberry polyphenol extract and fructo-oligosaccharides; HPP, control high-fat diet enriched with raspberry polyphenol extract and pectin. TNFα, tumour necrosis factor α; IL-6, interleukin 6; IL-10, interleukin 10.

The major parameters of lipid homeostasis are shown in Table 3 and Table 4. The strongest effect on lipid metabolism was observed in rats from the HPF group. Among all experimental groups, the TGs in the plasma and liver were considerably reduced in the HPF group compared to the control H group. Additionally, the level of liver cholesterol was the lowest in the HPF group. In rats from the HPF and HPP groups, the liver fat content was considerably decreased, whereas liver mass was increased in rats from the HPF group compared to those from the control H group. The experimental diets also affected molecular factors associated with lipid metabolism in the liver (Figure 1). A comparison of all experimental groups showed that the expression of PPARα and ANGPTL4 in the liver was lower in rats from the HP and HPF groups than in rats from the HPP group. Furthermore, in the HPF group observed the lowest liver expression of the PPARγ and SREBP1c. However, when compared to the control H group, the liver expressions of PPARα and ANGPTL4 were reduced only in rats from the HPF group, while PPARγ expression was considerably reduced in all experimental groups. Disturbances in liver lipid metabolism affected by an obesogenic diet might be associated with observed changes in selected parameters of oxidative stress and inflammation (Table 4 and Figure 2). In comparison to the control H group, only the HP and HPF groups showed reduced MDA levels in the liver. Moreover, compared with rats from the control H group, rats from the HP and HPF groups showed considerable decreased AST plasma levels, but the decrease was more pronounced in the HPF group. Among all examined groups, the plasma level of IL-6 was the lowest in the HP and HPF groups.

**Figure 3:**
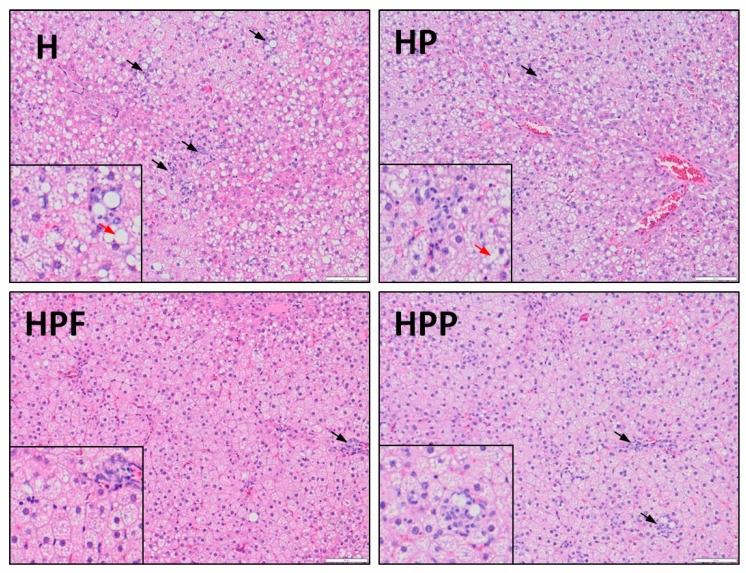
Hepatic histology in rats fed the experimental diets. H, control high-fat diet; HP, control high-fat diet enriched with raspberry polyphenol extract; HPF, control high-fat diet enriched with raspberry polyphenol extract and fructo-oligosaccharides; HPP, control high-fat diet enriched with raspberry polyphenol extract and pectin. (H) Liver with steatosis. Several inflammatory foci within the hepatic lobule (black arrow) and enhanced hepatocyte ballooning (red arrow). (HP) Reduced amount of inflammatory cells (black arrow) and mild ballooning of cells. Hepatocytes have rounded contours with clear reticular cytoplasm (red arrow). (HPF) There is one area of inflammatory cells (black arrow), the cytoplasm is pink and granular, and liver cells have sharp angles. (HPP) There are several inflammatory foci within the hepatic lobule (black arrow), and the shape of liver cells is quite similar to that observed in the HPF group. The liver samples were stained with haematoxylin and eosin at 20× and 40× (small image) magnification.

Changes in lipid metabolism and inflammatory factors were also observed in the analyses of liver histological characteristics (Table 5, Figure 3). The liver histological preparations from the control H group showed that an obesogenic diet promoted the development of NAFLD by increasing steatosis, hepatocyte ballooning, lobular and portal inflammation. All experimental groups with RE showed mitigated NAFL-related disorders to different extents. In the HP group, hepatocyte ballooning and lobular and portal inflammation were considerably reduced, but the decrease was more pronounced in the HPF and HPP groups. In these groups, the stages of steatosis were also significantly lower than those in the H group. Among the experimental groups, the most favourable effects on liver lobular and portal inflammation were reported in the HPF group.

## 4. Discussion

The results of the present study indicated that RE obtained from raspberry pomace was a valuable source of polyphenols and consisted of 47.8 g/100 g phenolic compounds, most of which were sanguiin H-6, lambertianin C (ellagitannins), proanthocyanidins (flavanols), cyanidin-3-o-spohoroside and cyaniding-3-o-glucoside (anthocyanins). Other studies on polyphenolic raspberry extracts have reported similar polyphenol profiles [6]; however, the range of the total phenolic contents is very wide, from 37.6 to 63.3 g/100 g [9,27,28]. The profiles and contents of these bioactive substances in extracts from raspberries are strongly influenced by genotypic and extrinsic factors, such as agricultural practices, seasonal differences, developmental stage and extraction methods [27].

Supplementation with raspberry polyphenols may play important roles in the regulation of bacterial fermentation processes in the hindgut and liver lipid metabolism [10,28,29]. These polyphenols interact with intestinal microbiota [30] and thus affect their activity and the production of SCFAs. The SCFAs produced by intestinal microbiota exert protective effects on metabolic disorders [31]; however, some studies also report that these fatty acids might promote the development of obesity and thus NAFL-related disorders [32,33]. Current nutritional studies show that obesity might be associated with high levels of SCFAs but not gut microbiota richness at the phylum level [34]. In this study, the addition of the RE to the obesogenic diet had no effect on the production of bacterial SCFAs; however, when polyphenolic extract was added together with the examined nondigestible saccharides, especially FOSs, the concentration of SCFAs was considerably reduced in the caecum. Our previous study on rats [14] showed that the combination of strawberry polyphenols and FOSs increased the metabolism of ellagitannins and thus affected the activity of the microbiota by lowering the production of SCFAs in the caecum. Furthermore, the mechanisms affecting the bacterial fermentation process in the gastrointestinal tract might be associated with the absorbed polyphenols, which might be secreted together with bile acids into the intestine [11]. Some studies have reported that ellagitannins may decrease enterobacterial growth or exert antimicrobial effects against probiotics [30,35]. Additionally, anthocyanins might have antibacterial properties against bacteria living in the gastrointestinal tract [29,36]. Moreover, the combination of RE with both of the nondigestible saccharides favourably reduced the bacterial production of PSCFAs in the caecum and increased the mass of caecal tissue. This effect might be related to decreased anaerobic bacterial polypeptide and amino acid fermentation [37]. In addition to regulatory effects of raspberry polyphenols, the supplementation with FOSs and PECs might also exert effects on the intestinal microbiota activity and lipid metabolism. These nondigestible saccharides have different chemical structures and thus can affect the balance of gut microbiota to a different extent. The feeding experiment on mice fed with different mixtures of nondigestible saccharides showed that FOSs increased the SCFA production more effectively than PECs [13]. In the present study, however, an opposite effect was observed, therefore it might be assumed that the raspberry polyphenols considerably modulated effect of examined nondigestible saccharides. Nakamura et al. [38] in their study on mice showed that the dietary addition of FOSs (2.5%) either inhibited the uptake of monoglycerides or nonesterified fatty acids in the small intestine, or altered gastrointestinal function related to fat absorption, thus reducing the plasma TGs levels and affected lipid metabolism in the liver. Additionally, dietary supplementation with PECs (10%) can regulate lipid metabolism through gel formation and binding the bile acids and micelle components in the gut, such as monoglycerides, free fatty acids and cholesterol [39]. In this experiment, only supplementation with FOSs and raspberry polyphenols considerably reduced the plasma TGs. Such an effect might be associated with the different dietary amount of nondigestible saccharides used in the present study (3%). Apart from local intestinal effects, supplementation with raspberry polyphenols and examined nondigestible saccharides favourably mitigated the liver metabolic disorders caused by an obesogenic diet. Currently, the accepted pathophysiological model for NAFLD is the “two hits” model. The first stage is associated with the accumulation of fat and TGs in the liver as a result of changes in the influx, synthesis, oxidation and transport of fatty acids. The second stage, triggered by the first hit, includes oxidative imbalance and induction of proinflammatory cytokines as a result of mitochondrial dysfunction, lipid peroxidation and activation of inflammatory pathways [40,41]. In this study, the combination of FOSs and RE exerted the most favourable effect on NAFL-related disorders. This diet considerably reduced plasma TGs, liver cholesterol, TGs and fat content. Moreover, histological analyses showed that this dietary combination exerted the most favourable effect on liver through the reduction of liver steatosis and hepatocyte ballooning. The observed effects might be partially related to considerably decreased expression of ANGPTL4 and PPARγ in the liver. ANGPTL4 is responsible for the inhibition of lipoprotein lipase (LPL), thereby regulating the hydrolysis of lipoprotein and TGs and the uptake of fatty acids into tissue [42], whereas hepatic PPARγ expression promotes lipid uptake, triacylglycerol storage, and lipid droplet formation [43]. A similar effect was observed in a nutritional study on mice fed a high-fat diet [28], where daily oral administration of raspberry polyphenols upregulated hepatic LPL and reduced fat accumulation. Another gene that is also important in the regulation of liver lipid metabolism is PPARα. This gene is linked with mechanisms of liver fatty acid catabolism [43]. However, supplementation with raspberry polyphenols and FOSs reduced the hepatic expression of PPARα; therefore, it might be assumed that the main mechanisms responsible for the regulation of NAFL-related disorders in rats from HPF group are associated with the downregulation of PPARγ and ANGPTL4. 

The disorders caused by an obesogenic diet affect numerous hepatic lipid pathways, consequently increasing hepatic inflammation, oxidative stress and fibrosis [44]. Favourable effects of RE on liver lipid metabolism may also explain mitigation of the liver inflammatory processes and lipid peroxidation caused by an obesogenic diet. Diets with RE alone or with FOSs lowered the level of plasma IL-6, the activity of AST and the level of MDA in the liver. Furthermore, the most effective reductions in liver lobular and portal inflammation were observed when the diet was enriched with RE and FOSs. Kang et al. [45], in a nutritional study on C57BL/6 male mice fed a high-fat diet, also showed that the addition of ellagitannins from raspberry seed flour to the diet significantly decreased hepatic oxidative stress and thus reduced proinflammatory gene expression and macrophage infiltration.

## 5. Conclusions

This nutritional study showed that the effect of raspberry polyphenols against NAFL-related disorders might be enhanced with FOSs and PECs. It was reported that this dietary combination considerably reduced microbial fermentation in the caecum. Among the examined nondigestible saccharides, the combination of FOSs with RE exerted the most favourable effects on lipid metabolism and thus inflammatory processes and lipid peroxidation in the liver. The observed effects might be partially related to decreased hepatic expressions of PPARγ and ANGPTL4. Surprisingly, in the rats fed the diet with PECs and the examined extract, the expression levels of all examined genes regulating liver lipid metabolism were higher than in rats fed diet with FOSs and RE. These data suggest that the activity of raspberry polyphenols against liver disorders caused by an obesogenic diet might be driven by supplementation with specific dietary fibres. Collectively, the results indicated that the dietary combination of RE with FOSs exerted more favourable effects than the combination with PECs; therefore, dietary addition of RE together with FOSs might be considered a dietary compound that effectively mitigates NAFL-related disorders induced by an obesogenic diet.

## Figures and Tables

**Table 1:** Polyphenol composition of raspberry extract.

Compound	mg/100 g
Ellagitannins (ET) *n* = 3	
Lambertianin C	18,314.0 ± 1172.6
Lambertianin C minus ellagic acid moiety 1 ^a^	764.1 ± 28.8
Lambertianin C minus ellagic acid moiety 2 ^a^	196.4 ± 10.9
Lambertianin C minus ellagic acid moiety 3 ^a^	426.6 ± 6.7
Sanguiin H-6	16,975.8 ± 350.4
Sanguiin H-6 minus gallic acid moiety ^b^	221.6 ± 9.7
Sanguiin H-6 plus gallic acid moiety ^b^	356.3 ± 22.4
Sanguiin H-10 isomer 1 ^b^	466.4 ± 14.0
Sanguiin H-10 isomer 2 ^b^	533.0 ± 20.7
Sanguiin H-10 isomer 3 ^b^	301.2 ± 10.7
Ellagic acid pentose conjugate ^c^	171.7 ± 11.4
Ellagic acid	196.7 ± 18.1
Total ET	38,088.9 ± 1503.6
Total EAC	368.3 ± 29.4
Total ET + EAC	38,923.6 ± 1547.0
Flavanols (FLAVA) *n* = 3	
Total FLAVA	8371.7 ± 486.5
(+)-Catechin	208.0 ± 5.4
(−)-Epicatechin	343.5 ± 5.5
Proanthocyanidins	7820.2 ± 475.7
Extension units (%)	
(+)-Catechin	28.5 ± 0.1
(−)-Epicatechin	3.0 ± 0.0
Terminal units (%)	
(+)-Catechin	13.5 ± 0.0
(−)-Epicatechin	55.0 ± 0.1
mDP (°)	1.5 ± 0.0
Anthocyanins (ACY) *n* = 3	
Cyanidin-3-*O*-spohoroside ^d^	314.6 ± 11.5
Cyanidin-3-*O*-glucosyl-rutinoside ^d^	27.0 ± 0.7
Cyanidin-3-*O*-glucoside	152.0 ± 0.9
Cyanidin-3-*O*-rutinoside ^d^	11.5 ± 0.1
Pelargonidin-3-*O*-glucoside ^d^	4.5 ± 0.3
Total ACY	509.6 ± 10.9
Total polyphenols (TPH)	47,804.8 ± 1060.5

Values are expressed as the mean ± standard deviation (mg/100 g); *n*, number of measurements; mDP, mean degree of polymerisation; total EAC (total content of ellagic acid), the total content of ellagic acid and its conjugates. ^a^ The content of these substances was calculated based on the lambertianin C standard. ^b^ The content of these substances was calculated based on the sanguiin H-6 standard. ^c^ The contents of these substances were calculated based on ellagic acid standards. ^d^ The content of anthocyanins was calculated based on the cyanidin-3-O-glucoside standard.mDP: mean degree of polymerisation.

**Table 2:** Growth parameters and basic indicators of gastrointestinal tract function in the rats fed the experimental diets.

Parameters	Groups				ANOVA *p* Value
	H	HP	HPF	HPP	
Diet intake (g/day)	25.32 ± 2.11	24.82 ± 1.56	23.92 ± 2.88	25.02 ± 1.31	NS
Final body weight (g)	466.64 ± 11.91	472.74 ± 14.22	465.43 ± 9.21	457.94 ± 15.37	NS
Final body fat (g)	137.62 ± 4.42	142.69 ± 9.98	130.05 ± 6.46	131.48 ± 5.44	NS
Final body lean (g)	253.43 ± 6.82	254.48 ± 5.53	258.05 ± 5.19	254.96 ± 6.65	NS
Epididymal fat (g)	20.54 ± 1.37	21.62 ± 2.17	18.64 ± 1.45	19.31 ± 1.37	NS
Small intestine:					
Tissue mass (g)	6.14 ± 0.35	6.62 ± 0.19	6.99 ± 0.27	6.72 ± 0.16	NS
pH	7.01 ± 0.08	6.83 ± 0.08	6.81 ± 0.078	6.71 ± 0.10	NS
Caecum:					
Tissue mass (g)	0.56 ± 0.03 ^b^	0.62 ± 0.02 ^ab^	0.69 ± 0.03 ^a^	0.66 ± 0.02 ^a^	<0.01
pH	7.08 ± 0.08 ^a^	6.91 ± 0.09 ^ab^	7.08 ± 0.08 ^a^	6.70 ± 0.13 ^b^	<0.05
SCFA (µmol/g digesta)					
Acetic acid	26.38 ± 1.85 ^a^	27.13 ± 4.12 ^a^	17.28 ± 1.71 ^b^	21.95 ± 1.79 ^ab^	<0.05
Propionic acid	7.84 ± 0.34	8.03 ± 0.41	5.83 ± 0.60	6.95 ± 0.87	0.054
*Iso*-butyric acid	0.63 ± 0.04 ^a^	0.60 ± 0.07 ^ab^	0.44 ± 0.04 ^b^	0.40 ± 0.06 ^c^	<0.05
Butyric acid	1.86 ± 0.19 ^a^	1.80 ± 0.24 ^a^	0.93 ± 0.17 ^b^	1.52 ± 0.12 ^a^	<0.01
*Iso*-valeric acid	0.78 ± 0.05 ^a^	0.78 ± 0.06 ^a^	0.57 ± 0.05 ^b^	0.55 ± 0.06 ^b^	<0.01
Valeric acid	0.62 ± 0.03 ^a^	0.58 ± 0.05 ^ab^	0.44 ± 0.05 ^b^	0.26 ± 0.05 ^c^	<0.001
SCFA profile (%)					
Acetic acid	68.90 ± 0.95	67.92 ± 2.72	67.64 ± 1.33	69.63 ± 1.87	NS
Propionic acid	20.82 ± 0.85	21.79 ± 1.71	22.84 ± 1.19	21.81 ± 1.69	NS
Butyric acid	4.85 ± 0.37	4.66 ± 0.40	3.60 ± 0.54	4.90 ± 0.32	NS
Total PSCFAs	2.03 ± 0.10 ^a^	1.98 ± 0.16 ^a^	1.48 ± 0.15 ^ab^	1.22 ± 0.18 ^b^	<0.01
Total SCFAs	38.12 ± 2.24 ^a^	38.94 ± 4.62 ^a^	25.53 ± 2.38 ^b^	31.64 ± 2.65 ^ab^	<0.05

Values are the mean ± SEM; *n* = 8. ^a,b,c^ Mean values not sharing the same superscript letter within a row (a, b or c) are different at *p* < 0.05 in a post hoc test. (Dunn’s test). H, control high-fat diet; HP, control high-fat diet enriched with raspberry polyphenol extract; HPF, control high-fat diet enriched with raspberry polyphenol extract and fructo-oligosaccharides; HPP, control high-fat diet enriched with raspberry polyphenol extract and pectin. PSCFAs, putrefactive short-chain fatty acids (the sum of iso-butyric, iso-valeric and valeric acids); SCFAs, short-chain fatty acids.

**Table 3:** Plasma lipid profile of rats fed the experimental diets.

Parameters	Groups				ANOVA *p* Value
	H	HP	HPF	HPP	
TG (mmol/L)	1.05 ± 0.10 ^a^	0.89 ± 0.11 ^a^	0.64 ± 0.05 ^b^	0.70 ± 0.06 ^a^	<0.05
TC (mmol/L)	2.99 ± 0.24	2.69 ± 0.20	2.97 ± 0.21	2.40 ± 0.30	NS
HDL (mmol/L)	0.42 ± 0.02	0.44 ± 0.02	0.39 ± 0.03	0.36 ± 0.03	NS
non-HDL (mmol/L)	2.58 ± 0.25	2.25 ± 0.20	2.59 ± 0.22	2.04 ± 0.31	NS

Values are the mean ± SEM; *n* = 8. HDL, HDL-cholesterol, LDL, LDL-cholesterol; TC, total cholesterol; TG, triacylglycerols. ^a,b^ Mean values not sharing the same superscript letter within a row (a or b) are different at *p* < 0.05 in a post hoc test. (Dunn’s test). H, control high-fat diet; HP, control high-fat diet enriched with raspberry polyphenol extract; HPF, control high-fat diet enriched with raspberry polyphenol extract and fructo-oligosaccharides; HPP, control high-fat diet enriched with raspberry polyphenol extract and pectin.

**Table 4:** Liver indicators of inflammation, oxidative stress and lipid metabolism in rats fed the experimental diets.

Parameters	Groups				ANOVA *p* Value
	H	HP	HPF	HPP	
Liver (g)	15.93 ± 0.50 ^b^	17.10 ± 0.75 ^ab^	18.71 ± 0.51 ^a^	16.21 ± 0.71 ^b^	<0.05
Fat content (%)	34.45 ± 1.47 ^a^	31.13 ± 1.87 ^a^	27.25 ± 1.24 ^b^	27.57 ± 1.27 ^b^	<0.01
Cholesterol (mg/g liver)	10.38 ± 0.41 ^a^	8.73 ± 1.02 ^a^	7.63 ± 0.76 ^b^	8.54 ± 0.99 ^a^	<0.05
TG (mg/g liver)	19.42 ± 0.85 ^a^	16.70 ± 1.80 ^ab^	14.81 ± 0.48 ^b^	17.75 ± 1.03 ^ab^	<0.05
MDA (ng/g liver)	781.1 ± 25.4 ^a^	680.6 ± 21.0 ^b^	642.5 ± 16.9 ^b^	752.1 ± 35.9 ^a^	<0.01
Plasma indicators (U/L)					
AST	204.1 ± 12.7 ^a^	141.9 ± 18.9 ^bc^	134.6 ± 8.9 ^c^	167.6 ± 25.2 ^ab^	<0.05
ALT	122.2 ± 45.2	121.7 ± 34.1	108.7 ± 27.0	97.1 ± 29.5	NS
ALP	92.8 ± 8.8	85.6 ± 10.5	104.2 ± 3.9	89.7 ± 5.2	NS

Values are the mean ± SEM; *n* = 8. AST, aspartate transaminase; ALT, alanine transaminase; ALP, alkaline phosphatase; MDA, malondialdehyde; TG, triglyceride. ^a,b,c^ Mean values not sharing the same superscript letter within a row (a, b or c) are different at *p* < 0.05 in a post hoc test. (Dunn’s test). H, control high-fat diet; HP, control high-fat diet enriched with raspberry polyphenol extract; HPF, control high-fat diet enriched with raspberry polyphenol extract and fructo-oligosaccharides; HPP, control high-fat diet enriched with raspberry polyphenol extract and pectin.

**Table 5:** Liver histological characteristics of rats fed the experimental diets.

Parameters		Stage ^1^		ANOVA *p* Value
	Group	Low	High	
Steatosis, *n* (%)	H ^a^	0 (0)	8 (100)	<0.01
	HP ^a^	2 (25)	6 (75)	
	HPF ^b^	7 (87.5)	1 (12.5)	
	HPP ^b^	6 (75)	2 (25)	
Ballooning, *n* (%)	H ^a^	0 (0)	8 (100)	<0.01
	HP ^b^	4 (50)	4 (50)	
	HPF ^c^	8 (100)	0 (0)	
	HPP ^c^	8 (100)	0 (0)	
Lobular inflammation, *n* (%)	H ^a^	0 (0)	8 (100)	<0.05
	HP ^b^	3 (37.5)	5 (62.5)	
	HPF ^c^	7 (87.5)	1 (12.5)	
	HPP ^ab^	2 (25)	6 (75)	
Portal inflammation, *n* (%)	H ^a^	1 (12.5)	7 (87.5)	<0.05
	HP ^b^	3 (37.5)	5 (62.5)	
	HPF ^c^	6 (75)	2 (25)	
	HPP ^bc^	4 (50)	4 (50)	

*n* = 8 in each group. ^a,b,c^ Mean values not sharing the same superscript letter (a, b or c) are different at *p* < 0.05 in a post hoc test (Dunn’s test). H, control high-fat diet; HP, control high-fat diet enriched with raspberry polyphenol extract; HPF, control high-fat diet enriched with raspberry polyphenol extract and fructo-oligosaccharides; HPP, control high-fat diet enriched with raspberry polyphenol extract and pectin. ^1^ Low = 0 or 1 stage; High = 2 or 3 stage.

## Data Availability

Data is available from the corresponding authors upon reasonable request.

## Supplementary Materials

The following are available online at https://www.mdpi.com/2072-6643/13/3/833/s1, Table S1: Composition of the group-specific diets.
