# Supplementary material for: Fructo-Oligosaccharides and Pectins Enhance Beneficial Effects of Raspberry Polyphenols in Rats with Nonalcoholic Fatty Liver
Source: Nutrients. 2021 Mar 3;13(3):833. doi: 10.3390/nu13030833 (PMC8001257; doi:10.3390/nu13030833)
Supplement: Supplementary file 1 [file nutrients-13-00833-s001.pdf]

Table S1. Composition of the group-specific diets

(g/100 g diet)	Groups			
	H	HP	HPF	HPP
Casein	20	20	20	20
DL-methionine	0.3	0.3	0.3	0.3
Rapeseed oil	2	2	2	2
Cholesterol	1	1	1	1
Lard	23	23	23	23
Polyphenolic extract	-	0.64	0.64	0.64
Fructooligosaccharides	-	-	3	-
Pectin	-	-	-	3
Saccharose	10	10	10	10
Cellulose	3	3	3	3
Corn starch	36	35.36	32.36	32.36
Mineral mix[1]	3.5	3.5	3.5	3.5
Vitamin mix[1]	1	1	1	1
Choline chloride	0.2	0.2	0.2	0.2

H, control high-fat diet; HP, control high-fat diet enriched with raspberry polyphenol extract; HPF, control high-fat diet enriched with raspberry polyphenol extract and fructooligosaccharides; HPP, control high-fat diet enriched with raspberry polyphenol extract and pectin.

[1] Recommended level for the AIN-93G diet.

Supplemental Table 2. Basic chemical composition of raspberry polyphenol extract.

Compound	[g/100 g]
Basic compounds	
Dry matter (AOAC 940.26)	94.79 ± 0.18
Protein (AOAC 920.152)	4.74 ± 0.45
Fat (AOAC 930.09)	0.51 ± 0.04
Ash	2.11 ± 0.06
TDF (AOAC 985.29; 991.42)	0.00 ± 0.00
IDF	0.00 ± 0.00
SDF	0.00 ± 0.00

TDF, Total dietary fibre; ISD, insoluble dietary fibre SDF, soluble dietary fibre.
AOAC - Horwitz W, Latimer G.W. (2007) Official methods of analysis of the AOAC International, 2005, 18th edn. AOAC International, USA.
